# A 10-Year Review in the Trends in the Operative Management and Timing of Resection in Pediatric Congenital Airway Malformations: An ACS NSQIP-Pediatric Study

**DOI:** 10.3390/children13050688

**Published:** 2026-05-17

**Authors:** Marc M. Mankarious, Alicia C. Greene, Olivia Ziegler, Swetha Jayavelu, Anthony Y. Tsai, Robert L. Ricca, Afif N. Kulaylat

**Affiliations:** 1 Department of Surgery, Penn State Hershey Medical Center, Hershey, PA 17033, USA; mmankarious@pennstatehealth.psu.edu (M.M.M.); agreene6@pennstatehealth.psu.edu (A.C.G.); oziegler@pennstatehealth.psu.edu (O.Z.); sjayavelu@pennstatehealth.psu.edu (S.J.); 2 Division of Pediatric Surgery, Penn State Children’s Hospital, Hershey, PA 17033, USA; atsai1@pennstatehealth.psu.edu (A.Y.T.);

**Keywords:** congenital pulmonary airway malformation (CPAM), congenital lung malformation (CLM), video-assisted thoracoscopic surgery (VATS), pediatric surgery, minimally invasive surgery

## Abstract

**Highlights:**

**What are the main findings?**

- Thoracoscopic (VATS) resection for pediatric CPAM increased markedly, becoming the predominant surgical approach.
- Despite advocacy for early intervention, the timing of surgery remained variable, with most resections performed after six months of age and no significant reduction in postoperative complications by age group.

**What are the implications of the main findings?**

- Current practice patterns favor minimally invasive surgery but do not reflect a consistent shift toward earlier resection of asymptomatic CPAM.
- These findings highlight ongoing debate regarding optimal timing of surgery and underscore the need for prospective studies to guide evidence-based recommendations.

**Abstract:**

**Background/Objectives**: The optimal timing of asymptomatic congenital pulmonary airway malformations (CPAM) is controversial. Early resection may reduce inflammation and scarring secondary to respiratory infections, but contemporary practice patterns are unknown. This study assesses trends in operative timing and approach over the past decade. **Methods**: A retrospective review was performed of 1934 CPAM patients in NSQIP-P undergoing resection (2012–2021). Trends in surgical approach and age at resection were assessed using Mann–Kendall tests. Multivariable logistic and linear regression were used to model the influence of age at operation on operative length, postoperative complications, and postoperative length of stay. **Results**: Thoracoscopic approach increased from 47.2% in 2012 to 80.8% in 2021 (*p* < 0.001). Median age at operation was 7.7 months. There was a downtrend in the open approach in patients ≤3 months old (tau = −0.511, *p* < 0.05) without a corresponding increase in VATS approach (tau = −0.11, *p* = 0.72). Instead, there was a statistically significant uptrend in all other age cohorts >3 months old in the VATS approach. After adjusting for confounders there was no difference in complication rates between age cohorts. **Conclusions**: Adoption of thoracoscopic resection for CPAM has substantially increased. Despite the reported benefits of earlier resection, the timing of surgical resection remains variable with most surgeries still occurring after six months of age. Additionally, the decline in open surgeries in patients ≤3 months may reflect a preference towards the VATS approach in a slightly older infant population. Further research is necessary to determine optimal timing for CPAM resection.

## 1. Introduction

Congenital pulmonary airway malformations (CPAMs) are developmental anomalies of the bronchial tree that result in abnormal proliferation of lung tissue [1]. With advances in antenatal imaging and screening programs, an increasing number of CPAMs are detected annually with an estimated annual incidence of 1 in 7200 live births [2,3]. While immediate resection may be required in CPAMs causing respiratory distress or compromise, the majority of lesions are asymptomatic with significant variability in subsequent management [4]. In North America, elective resection is recommended for asymptomatic patients as these lesions are prone to infection, may represent an occult malignancy, or can potentially degenerate into malignancy later in life [5,6].

The optimal timing for surgery remains controversial. Advocates of early operative intervention in patients describe shorter operative times secondary to less scarring and inflammation encountered at the dissection of the fissure, with a comparable morbidity profile [7,8,9]. Conversely, others prefer delaying surgery until the infant is older, noting larger infant size and associated working domain. Furthermore, some studies have challenged the notion that early surgical intervention is superior given increased transfusion rates, longer hospital length of stay (LOS), longer pleural drainage, and lower rates of video-assisted thoracoscopic surgery (VATS) approach in those patients compared to older cohorts [10,11].

The objective of this study was to evaluate national trends in operative timing and surgical approach for CPAM and to assess the association between age at resection and postoperative outcomes. We hypothesize that VATS utilization would increase over time, with a greater proportion of surgeries performed in younger patients.

## 2. Materials and Methods

### 2.1. Data and Population

Data was collected from the American College of Surgeons National Surgical Quality Improvement Program Pediatric (ACS NSQIP-P) Participant Use Data File from 2012 to 2021. This data set contains information for pediatric surgery cases collected from 150 participating hospitals. The database contains patient-level, aggregate data abstracted by a trained clinical reviewer, which includes clinical variables from individual patient records such as preoperative risk factors and intraoperative details, in addition to clinical outcomes such as 30-day postoperative morbidity and mortality. As NSQIP-P is a procedure-based database, only patients undergoing operative intervention are captured; patients managed non-operatively are not included [12].

Patients less than 18 years of age diagnosed with CPAM were identified using the International Classification of Diseases, Ninth Revision (ICD-9) diagnosis codes: 748.4, 748.5, 748.69, and Tenth Revision (ICD-10) diagnosis codes: Q33.0, Q33.9, Q33.8, Q33, Q33.3, Q33.6. Patients with bronchopulmonary sequestration or congenital lobar emphysema were excluded. Using Current Procedural Terminology (CPT) codes, patients were stratified based on open thoracotomy (32480, 32505, 32484, 32140, 32482, 32608, 32097) or VATS (32663, 32666, 32669, 32670, 32655). CPT codes were further used to categorize the extent of surgical resection, which included wedge resection (32666, 32505, 32140, 32608, 32097, 32655), segmentectomy (32669, 32484), single lobectomy (32663,32480), and bilobectomy (32482, 32670). The patients that underwent VATS were reviewed for any concurrent codes indicating an open thoracotomy (CPT: 32480, 32505, 32484, 32140, 32482, 32608, 32097) to identify the need for conversion. Urgent and emergent cases were excluded.

### 2.2. Covariates and Outcomes

Patient demographics and operative details were abstracted from the NSQIP-P database. Demographic data included age, sex, race, and Hispanic ethnicity. Age was adjusted for prematurity and then separated into age-based cohorts (≤3, >3 to ≤6, >6 to <12, and ≥12 months old). Patients undergoing surgical intervention prior to 40 weeks of gestational age were considered outliers (*n* = 62) and were excluded from this study. This small population raised the potential for an uncaptured confounder or coding error (e.g., in diagnosis or case urgency) as elective resection of CPAMs is relatively unusual in this age group. The covariates studied were derived from the clinical variables available in NSQIP-P and included weight at the time of surgery in kilograms (Kg) and the American Society of Anesthesiologists (ASA) classification. Preoperative risk factors occurring within 30 days of the principal surgery and comorbidities were captured and included: history of prematurity, neurological (seizure disorder, intraventricular hemorrhage, impaired cognitive status, cerebral palsy, or neuromuscular disorder), cardiac risk factors, gastrointestinal, hematologic or bleeding disorders, immunosuppressive (immune disease, organ transplant, chronic steroid use, chemotherapy, radiotherapy, or malignancy), and nutritional (recent weight loss or need for nutritional support). Only comorbidities and preoperative conditions with occurrences are described and included in the analysis. Intraoperative variables included wound classification (clean, clean-contaminated, contaminated, or dirty), conversion to an open approach, and operative time. Operative time refers to the surgeon’s start and stop times.

Outcomes of interest were (1) the proportion of VATS cases over time, (2) operative approach stratified by age over time, and (3) clinical outcomes. Clinical outcomes included early postoperative complications, postoperative LOS, reoperation, and readmission. Postoperative complications are defined as the presence of any NSQIP-P complication within 30 days of operation unless otherwise noted. Postoperative complications included surgical site infection (superficial, deep, or organ space), pneumonia, urinary tract infection (UTI), cardiac arrest, seizure, bleeding or transfusion requirement, and sepsis.

### 2.3. Statistical Analysis

Mann–Kendall test was used to assess trends over time whereas the Kendall rank correlation coefficient (tau) was used to determine the direction of the trend with negative tau values correlating to a downtrend and positive values correlating to an uptrend. Patient characteristics, intraoperative variables, and clinical outcomes were stratified by age group and were compared using Pearson’s chi-square tests and Fisher’s exact tests, as appropriate.

Multivariable logistic regression was used to analyze any postoperative complications with results reported as odds ratios (OR) and 95% confidence intervals (CI). Variables that were statistically significant in univariable analysis were used as covariables in multivariable analysis and included age, weight at operation, race, ethnicity, approach, extent of resection, cardiac comorbidities, nutritional comorbidities, and wound classification. Multivariable linear regression was used to analyze operative time and postoperative LOS after adjusting for the same aforementioned variables. All analyses were performed using R statistical software (version 4.2.0 Vienna, Austria). *p*-values less than 0.05 were considered statistically significant.

## 3. Results

### 3.1. Patient Characteristics

There were 1934 patients identified in NSQIP-P with CPAM who underwent an elective surgical resection. Baseline demographics and characteristics are listed in Table 1. The median patient age was 7.70 (IQR 5.00–13.07) months. Overall, patients were more likely to be male (55.2%), Caucasian (63.1%), and non-Hispanic (80.2%). Patients in ≤3 months old cohort were more likely to have cardiac comorbidities (22.7% vs. 12.5%, 12.1%, and 11.6%), nutritional comorbidities (12.2% vs. 0.8%, 1.2%, 1.5%), and ASA 4 classification (11.6% vs. 1.4%, 0.4%, 0.7%) compared to ages >3 to ≤6, >6 to <12, and ≥12 months old, respectively (*p* < 0.05 for all). VATS was less common in the ≤3 months old cohort occurring in 42.0% of patients compared to 70.9%, 69.4% and 66.4% in the >3 to ≤6, >6 to <12, and ≥12 months old cohorts, respectively (*p* < 0.05). The conversion rate to an open approach from VATS was similar in all cohorts.

### 3.2. Trends

**Figure 1:**
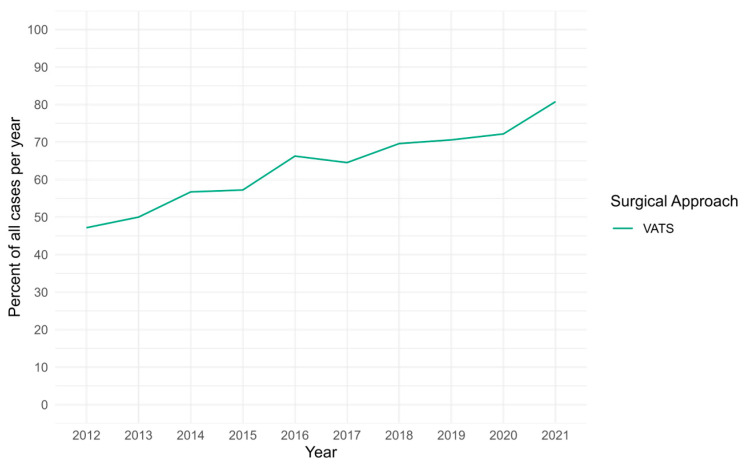
Trend in utilization of VATS as a proportion of total cases over the study interval for elective CPAM resection.

**Figure 2:**
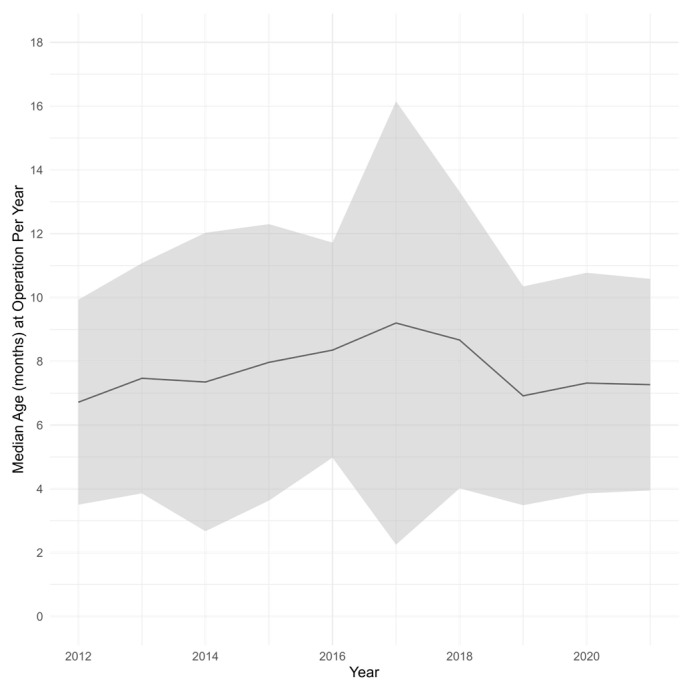
Median age in months at the time of pulmonary resection across the study interval. The gray band indicates the interquartile range.

To contextualize these baseline characteristics, we evaluated temporal trends in operative timing and surgical approach over the study interval. Overall, 1284 (66.4%) received a VATS resection. Over the study interval, the proportion of CPAM resections performed at the ≤3, >3 to ≤6, >6 to <12, and ≥12 months old groups were 9.4%, 25.2%, 37.7%, 27.7%, respectively (Table 1). Adoption of VATS for the overall cohort increased from 47.17% in 2012 to 80.78% in 2021 with a tau of 0.956 (*p* < 0.001) (Figure 1). The median age at operation by year remains largely unchanged, from 6.7 months in 2012 to 7.3 months in 2021 (tau = 0.067, *p* = 0.86) (Figure 2).

**Figure 3:**
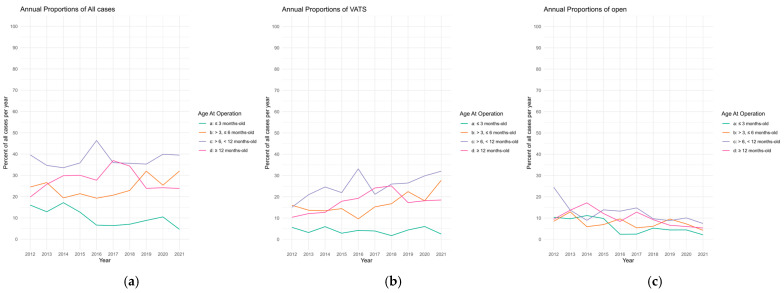
Trend for elective CPAM resection by age at the time of operation: (**a**) trend for timing of operation in all patients; (**b**) Trend for timing of operation in patients undergoing VATS; (**c**) trend for timing of operation in patients undergoing open thoracotomy. Each colored line represents the proportion of total cases that were performed in that age category over the study interval.

We analyzed the proportion of operations completed in each age-based cohort over the study interval stratified by approach (Figure 3). While all other cohorts had no statistically significant change in trend of age at operation, the proportion of operations in patients ≤3 months old decreased from 16.03% in 2012 to 4.6% in 2021 (tau = −0.511, *p* = 0.049) (Figure 3a). This downtrend in the overall cohort was coupled with an equivalent downtrend in the proportion of open operations in the ≤3 months old (tau = −0.511, *p* = 0.049). Expectedly, there was an overall statistically significant downtrend in the open operations in the >6 to <12 months old and the ≥12 months old cohorts (Figure 3b). The downtrend in the open approach in patients ≤3 months old did not have a corresponding uptrend in VATS approach (tau = −0.111, *p* = 0.721). Instead, there was a statistically significant uptrend in all other age cohorts >3 months old in the VATS approach (Figure 3c). Tau and *p*-values for Figure 3 are summarized in Table 2.

### 3.3. Operative Characteristics

Surgical details are summarized in Table 3. Operative time increased across cohorts from a median time of 136 min (IQR 95–172 min) in the ≤3 months old cohort to 182 min (IQR 131–242 min) in the ≥12-month-old cohort (*p* < 0.001).

### 3.4. Postoperative Outcomes

Postoperative outcomes are summarized in Table 3. Patients in ≤3 months old cohort were more likely to have bleeding requiring transfusion (11% vs. 7%, 3.4%, and 3.7%, *p* < 0.05) and any postoperative complication (17.7% vs. 12.3%, 9.7%, 11%, *p* < 0.05) compared to the >3 to ≤6, >6 to <12, and ≥12 months old cohorts, respectively. Remainder of outcomes including rates of SSIs, pneumonia, sepsis, reoperation, and unplanned readmissions were similar across all cohorts.

A multivariable logistic regression model for any postoperative complications, and bleeding requiring transfusion adjusted for age, weight, race, ethnicity, approach, extent of resection, ASA classification, cardiac comorbidities, nutritional comorbidities, and wound classification are summarized in Table 4.

The VATS approach had significantly lower odds of any complication (OR 0.69, 95% CI 0.51–0.94) and transfusion (OR 0.36, 95% CI 0.23–0.56) compared to open thoracotomy. Patients in ≤3 months old (OR 2.21, 95% CI 1.09–4.38) and >3 to ≤6 months old (OR 2.20, 95% CI 1.28–3.83) cohorts had higher odds of requiring transfusion compared to patients in the >6 to <12 months old cohort.

A multivariable linear regression for operative time and postoperative LOS that included age-based cohorts and adjusted for the aforementioned variables is also summarized in Table 4. Patients undergoing surgery at ≤3 months old and >3 to ≤6 months old had shorter operative times by 42 and 13 min, respectively, compared to patients undergoing surgery at >6 to <12 months old. On the other hand, patients undergoing surgical resection at ≤3 months old had a postoperative LOS that is 1.3 days longer than patients in the >6 to <12 months old cohort.

## 4. Discussion

This study highlights the current trends and surgical practices in the management of pediatric CPAMs over a 10-year period from 2012 to 2021 in NSQIP-P. Although the trend in VATS adoption significantly increased over time, the median age at operation did not change significantly in the past decade despite increasing literature advocating earlier resection of CPAM in asymptomatic patients given the risk of developing respiratory symptoms with increasing age, which is associated with increasing risk of adhesive and inflammatory scar tissue and a potentially more challenging operation [5,7,8,9]. Analysis of trends by age and approach demonstrated a decrease in the proportion of patients undergoing open resection in the ≤3 months old age group without a concurrent increase in VATS adoption in the same group over time. Instead, there was an uptrend in VATS approach in all groups of patients >3 months old. One possible explanation for this pattern in trends is that surgeons are deferring operations until patients are >3 months old, and more likely to then utilize a VATS approach. Accordingly, with the increasing adoption of VATS and higher likelihood of performing VATS in patients >3 months old, there is an overall downtrend in patients undergoing resection at ≤3 months old with an increasing VATS adoption in other cohorts. Postoperative complication rates were comparable between the cohorts.

The increased adoption of the VATS approach for elective CPAM resection is likely multifactorial, including increased surgical training and preference in minimally invasive techniques, improved visualization, lower morbidity profile, and family preference [13]. Prior studies have demonstrated comparable or improved outcomes with VATS compared to open thoracotomy, including lower complication rates and improved musculoskeletal outcomes [12,14,15]. Although previous reports have shown the plausibility and efficacy of VATS in neonates and newborn infants, our study shows that only 42.0% of patients ≤3 months old underwent VATS approach, compared to 70.9%, 69.4%, and 66.4% in the >3 to ≤6, >6 to <12, and ≥12 months old groups, respectively [8,16].

The trends in timing of operation may offer insight into how and when many pediatric surgeons choose to approach CPAM. Our study’s median age at operation of 7.7 months is consistent with previously published data from a multi-institutional registry for children with operative congenital lung malformations where the median age at operation was 6.7 months [16]. The optimal surgical timing for asymptomatic CPAMs is a subject of ongoing debate. Some authors advocate for early operation in the first three months of life given reduced inflammation which is associated with shorter operative times [7,8,9]. Our study demonstrated shorter operative times in patients ≤3 months old when compared to patients in the >3 to ≤6, >6 to <12, and ≥12 months old cohorts (136 vs. 166, 174, 182, respectively, *p* < 0.05). However, there are conflicting data regarding associations between postoperative complications and age at operation, with some large database and meta-analytic studies suggesting increased transfusion rates and longer length of stay in younger patients, while others demonstrate no significant difference in complication rates across age groups [10,11]. In our study, patients ≤3 months old were more likely to require transfusion and demonstrated a trend toward longer postoperative length of stay compared to older cohorts. These findings, in the context of conflicting data in the literature, may contribute to the variability in timing of resection observed in current practice.

While the timing of operative intervention in our study was variable, and no increased complications were observed in ≥12-month cohort, there is data to suggest benefit to surgery prior to one year of age. In a meta-analysis of 168 patients with CPAM, 64.3% of patients managed non-operatively became symptomatic and required surgical intervention [6]. These symptomatic patients had a significantly increased risk of postoperative morbidity compared to non-symptomatic patients (OR = 4.59, 95% CI 1.40–15.11). Accordingly, as the average time of symptom onset in infants was noted to be 10 months in a systematic review of 41 reports with 1,070 patients, some authors recommend CPAM resection before 10 months old to minimize the risk of postoperative complications from patients becoming symptomatic [4]. This is supported by improved long-term outcomes, as radionuclide imaging at 1, 5, and 10 years after lobectomy for congenital cystic lung malformations noted that patients receiving resections at <1 year old had a lower mean transit time (a marker for air trapping) than patients who were older at the time of surgery at 5 and 10 years [17].

Our study was limited by its retrospective observational design and use of a national surgical database. Importantly, NSQIP-P captures only patients undergoing operative intervention; therefore, our findings reflect a selected cohort of surgically treated patients and may not be generalizable to all patients with CPAM, particularly those managed conservatively. The data is submitted by hospitals participating in NSQIP-P and does not represent a statistically valid, nationally representative sample. Additionally, the database lacks granular clinical detail regarding lesion-specific characteristics, including CPAM subtype, lesion size, radiographic features, and symptom status. The absence of detailed lesion classification may substantially impact interpretation of the findings, as differences in lesion subtype, extent of disease, and symptom burden may influence the timing of resection, likelihood of VATS utilization, and perioperative outcomes. The database is coder-dependent and subject to misclassification. Additionally, the database does not provide thoracic-specific variables such as length of pleural drainage or presence of postoperative air leaks which may influence operative strategy. Furthermore, NSQIP-P does not provide center-level data regarding institutional resources or surgeon expertise, including access to minimally invasive techniques such as VATS. Variability in availability or adoption of VATS across institutions, or over time, may have influenced the observed trends and represents a potential unmeasured confounder. Lastly, postoperative outcomes are only captured for 30 days so we are unable to assess the mechanism by which surgical approach or operative age may influence long-term outcomes or subsequent operations. Despite these limitations, the use of a national surgical database allowed us to evaluate the trend and associated outcomes of surgical approach and age at the time of operation in a large sample of pediatric CPAM patients over a 10-year period.

## 5. Conclusions

Over the past decade, there has been a substantial increase in the adoption of thoracoscopic approaches to CPAM. Despite the reported benefits of earlier resection, the timing of surgical resection remains variable with most surgeries still occurring after six months of age. Additionally, the decline in open surgeries in patients ≤ 3 may reflect a preference towards the VATS approach in a slightly older infant population. Further research is necessary to determine the optimal timing of resection in CPAM.

## Figures and Tables

**Table 1:** Patient characteristics.

Variable	Total	<3 Month Old	≥3 & <6 Months Old	≥6 & <12 Months Old	≥12 Months Old	*p*-Value
	*n* = 1934	*n* = 181	*n* = 488	*n* = 729	*n* = 536	
Age in Months (median [IQR])	7.70 [5.00, 13.07]	1.90 [0.90, 2.47]	4.65 [3.76, 5.37]	8.13 [7.00, 9.47]	22.27 [15.37, 62.73]	<0.001
Male (%)	1067 (55.2)	110 (60.8)	260 (53.3)	410 (56.2)	287 (53.5)	0.271
Race (%):						0.043
White	1220 (63.1)	119 (65.7)	339 (69.5)	440 (60.4)	322 (60.1)	
Black	202 (10.4)	13 (7.2)	41 (8.4)	81 (11.1)	67 (12.5)	
Asian	94 (4.9)	7 (3.9)	23 (4.7)	36 (4.9)	28 (5.2)	
Other	418 (21.6)	42 (23.2)	85 (17.4)	172 (23.6)	119 (22.2)	
Hispanic (%)	382 (19.8)	18 (9.9)	98 (20.1)	157 (21.5)	109 (20.3)	0.006
Weight at Surgery (median [IQR]) (kg)	8.18 [6.89, 10.08]	4.89 [4.01, 5.73]	6.89 [6.19, 7.57]	8.37 [7.50, 9.18]	12.12 [10.22, 19.40]	<0.001
ASA Classification (%):						<0.001
1	103 (5.3)	8 (4.4)	28 (5.7)	31 (4.3)	36 (6.7)	
2	1169 (60.4)	77 (42.5)	286 (58.6)	456 (62.6)	350 (65.3)	
3	627 (32.4)	75 (41.4)	167 (34.2)	239 (32.8)	146 (27.2)	
4	35 (1.8)	21 (11.6)	7 (1.4)	3 (0.4)	4 (0.7)	
Premature Birth (%)	212 (11.0)	25 (13.8)	54 (11.1)	70 (9.6)	63 (11.8)	0.356
Cardiac Comorbidities (%)	252 (13.0)	41 (22.7)	61 (12.5)	88 (12.1)	62 (11.6)	0.001
Gastrointestinal Comorbidities (%)	105 (5.4)	10 (5.5)	34 (7.0)	35 (4.8)	26 (4.9)	0.367
Neurological Comorbidities (%)	9 (0.5)	0 (0.0)	2 (0.4)	3 (0.4)	4 (0.7)	0.607
Hematologic Comorbidities (%)	23 (1.2)	3 (1.7)	7 (1.4)	5 (0.7)	8 (1.5)	0.461
Immunologic Comorbidities (%)	17 (0.9)	0 (0.0)	4 (0.8)	6 (0.8)	7 (1.3)	0.428
Nutritional Comorbidities (%)	43 (2.2)	22 (12.2)	4 (0.8)	9 (1.2)	8 (1.5)	<0.001
Wound Classification (%):						<0.001
Clean	734 (38.0)	55 (30.4)	197 (40.4)	257 (35.3)	225 (42.0)	
Clean/Contaminated	1167 (60.3)	123 (68.0)	285 (58.4)	468 (64.2)	291 (54.3)	
Contaminated	18 (0.9)	1 (0.6)	5 (1.0)	1 (0.1)	11 (2.1)	
Dirty/Infected	15 (0.8)	2 (1.1)	1 (0.2)	3 (0.4)	9 (1.7)	
VATS Approach (%)	1284 (66.4)	76 (42.0)	346 (70.9)	506 (69.4)	356 (66.4)	<0.001
Extent of Resection (%):						0.078
Single Lobectomy	1604 (82.9)	153 (84.5)	422 (86.5)	610 (83.7)	419 (78.2)	
Wedge Resection	185 (9.6)	15 (8.3)	35 (7.2)	67 (9.2)	68 (12.7)	
Segmentectomy	116 (6.0)	9 (5.0)	25 (5.1)	41 (5.6)	41 (7.6)	
Bilobectomy	29 (1.5)	4 (2.2)	6 (1.2)	11 (1.5)	8 (1.5)	
Conversion to Open (%)	12 (0.6)	0 (0.0)	4 (0.8)	5 (0.7)	3 (0.6)	0.676

IQR, interquartile range; ASA, American Society of Anesthesiologists physical status, VATS, video-assisted thoracic surgery.

**Table 2:** Tau and *p*-values for overall trends presented in Figure 3. Negative tau indicates downtrend, positive tau indicates uptrend, *p*-value <0.05 considered statistically significant.

Age Category	All		Open		VATS	
	Tau	*p-*Value	Tau	*p-*Value	Tau	*p-*Value
≤3 months-old	−0.511	0.049	−0.511	0.049	−0.111	0.721
>3 & ≤6 months-old	0.333	0.211	−0.289	0.283	0.556	0.032
>6 & <12 months-old	0.156	0.592	−0.511	0.049	0.644	0.012
≥12 months-old	−0.022	1.000	−0.644	0.012	0.556	0.032

**Table 3:** Univariable analysis of postoperative complications and outcomes analyzed by age-based cohorts.

Variable	Total	<3 Month Old	≥3 & <6 Months Old	≥6 & <12 Months Old	≥12 Months Old	*p*-Value
	*N* = 1934	*n* = 181	*n* = 488	*n* = 729	*n* = 536	
Superficial SSI (%)	10 (0.5)	0 (0.0)	2 (0.4)	5 (0.7)	3 (0.6)	0.689
Organ Space SSI (%)	16 (0.8)	2 (1.1)	2 (0.4)	10 (1.4)	2 (0.4)	0.159
Pneumonia (%)	21 (1.1)	0 (0.0)	5 (1.0)	10 (1.4)	6 (1.1)	0.464
UTI (%)	4 (0.2)	0 (0.0)	2 (0.4)	2 (0.3)	0 (0.0)	0.454
Cardiac Arrest (%)	5 (0.3)	0 (0.0)	2 (0.4)	1 (0.1)	2 (0.4)	0.661
Seizure (%)	5 (0.3)	0 (0.0)	0 (0.0)	3 (0.4)	2 (0.4)	0.446
Bleeding Requiring Transfusion (%)	99 (5.1)	20 (11.0)	34 (7.0)	25 (3.4)	20 (3.7)	<0.001
Sepsis (%)	15 (0.8)	2 (1.1)	2 (0.4)	9 (1.2)	2 (0.4)	0.238
Reoperation (%)	81 (4.2)	7 (3.9)	21 (4.3)	27 (3.7)	26 (4.9)	0.783
Unplanned Readmission (%)	96 (5.0)	3 (1.7)	21 (4.3)	45 (6.2)	27 (5.0)	0.075
Any Postoperative Complication (%)	222 (11.5)	32 (17.7)	60 (12.3)	71 (9.7)	59 (11.0)	0.024
Operative Time (median [IQR]) (Mins)	171.0 [121.2, 226.0]	136.00 [95.00, 172.00]	166.50 [117.75, 214.50]	174.00 [126.00, 231.00]	182.00 [131.00, 242.25]	<0.001
Postoperative LOS (median [IQR]) (Days)	3.00 [2.00, 4.00]	3.00 [2.00, 5.00]	2.00 [2.00, 4.00]	3.00 [2.00, 3.00]	3.00 [2.00, 4.75]	<0.001

SSI, surgical site infection; UTI, urinary tract infection; LOS, length of stay; IQR, interquartile range.

**Table 4:** Multivariable analysis of postoperative complications and outcomes.

	Any Complication			Transfusion			Operative Time (Mins)			Postoperative LOS (Days)		
	OR	95% CI	*p*-Value	OR	95% CI	*p*-Value	Beta	95% CI	*p*-Value	Beta	95% CI	*p*-Value
**Variable**							177	152, 203	<0.001	2.8	1.6, 4.0	<0.001
Age Category												
≤3 month old	1.43	0.85, 2.36	0.2	2.21	1.09, 4.38	0.024	−42	−56, −27	<0.001	1.3	0.63, 2.0	<0.001
>3 & ≤6 months old	1.33	0.91, 1.93	0.13	2.2	1.28, 3.83	0.004	−13	−23, −3.5	0.008	0.33	−0.12, 0.79	0.15
>6 & ≤12 months old	—	—		—	—		—	—		—	—	
>12 months old	1.15	0.76, 1.75	0.5	0.88	0.43, 1.75	0.7	1.7	−8.6, 12	0.7	0.19	−0.30, 0.68	0.4
Weight At Operation (kg)	1	0.99, 1.01	0.9	1.01	0.98, 1.03	0.4	0.83	0.44, 1.2	<0.001	0.05	0.04, 0.07	<0.001
Approach												
Open	—	—		—	—		—	—		—	—	
VATS	0.69	0.51, 0.94	0.016	0.36	0.23, 0.56	<0.001	0.38	−7.6, 8.4	>0.9	−0.97	−1.4, −0.59	<0.001
Extent of Resection												
Single Lobectomy	—	—		—	—		—	—		—	—	
Wedge Resection	0.89	0.53, 1.43	0.6	0.82	0.37, 1.63	0.6	−62	−75, −49	<0.001	−0.34	−0.95, 0.27	0.3
Segmentectomy	0.2	0.05, 0.54	0.007	0.33	0.05, 1.07	0.13	−40	−56, −25	<0.001	−0.38	−1.1, 0.36	0.3
Bilobectomy	2.08	0.82, 4.80	0.1	1.85	0.51, 5.24	0.3	23	−7.2, 54	0.13	1.9	0.38, 3.3	0.014
ASA Classification												
1	—	—		—	—		—	—		—	—	
2	1.2	0.59, 2.76	0.6	1.78	0.53, 11.1	0.4	−1.7	−18, 15	0.8	0.25	−0.54, 1.0	0.5
3	2.08	1.02, 4.81	0.061	3.16	0.94, 19.7	0.12	9.7	−7.7, 27	0.3	0.65	−0.17, 1.5	0.12
4	2.32	0.73, 7.43	0.2	3.48	0.65, 26.8	0.2	−20	−54, 14	0.2	3.9	2.3, 5.6	<0.001

VATS, video-assisted thoracic surgery; ASA, American Society of Anesthesiologists physical status. Variables adjusted for include race, ethnicity, cardiac comorbidities, nutritional comorbidities, and wound classification as part of the multivariable analysis.

## Data Availability

The data that support the findings of this study are available from the American College of Surgeons National Surgical Quality Improvement Program-Pediatric (NSQIP-P). Restrictions apply to the availability of these data, which were used under institutional agreement; therefore, the data are not publicly available. Data may be available from the corresponding author upon reasonable request and with permission of the American College of Surgeons.

## Abbreviations

The following abbreviations are used in this manuscript:

ASA	American Society of Anesthesiologists
CPAM	Congenital pulmonary airway malformation
CPT	Current Procedural Terminology
ICD 9/10	International Classification of Diseases 9 or 10
IQR	Interquartile range
KID	Kids Inpatient Database
LOS	Length of stay
NSQIP-P	National Surgical Quality Improvement Program Pediatric
OR	Odds ratio
UTI	Urinary tract infection
VATS	Video-assisted thoracoscopic surgery
